# Annotations

**Published:** 1890-02-15

**Authors:** 


### Annotations.

**Stitches of Progress.**

The "society gossip" of a newspaper does not often contain much of real interest, but the announcement that the trousseau of Miss Maud Rendel, Mr. Henry Gladstone's bride, was made by Mrs. Ernest Hart's workers at Donegal House for once justifies the journalist in meddling with private affairs. Not because Mr. Henry Gladstone is the son of his father. Not because the feminine mind delights in descriptions of embroidery and lace. But because the women who wrought these dainty garments were a few years ago untaught peasants who hardly knew how to hold a needle. They were quick-witted, maybe, but what were their wits worth when there was nothing to exercise them on? Some artistic skill, handed down by one unconscious generation after another since Ireland led the van of Europe in the arts of peace, might be hidden in their souls, but who was to draw it out and put it to practical account? They had neither tools, nor teaching, nor a market for their work when it was produced. Mrs. Ernest Hart has given them all three. She has made her voice heard both in England and Ireland. She spent her own money, to begin with; then she spent time and energy, that cannot easily be reckoned, in winning others to believe, as she did, in the possibilities of successful work that lay dormant in the untaught peasants of Connemara. Lastly, she induced the Government to give her a grant of £1,000 to aid her work. The account she has given of the way in which that money was spent proves that she is no mere well-meaning lady, kindly but indiscriminating; but a far-sighted woman, capable of no little business forethought and organising skill. The schools of needlework and embroidery established in districts almost unknown to English feet; the encouragement and development of the indigenous arts of knitting and weaving; the bestowal of further opportunities for study on those who showed marked talent; all these things imply comfort instead of poverty, interesting work instead of despairing in dangerous idleness, healthy self-dependence instead of beggary. The work begun does not end with this generation; it gives to at least one quarter of Ireland the chance of that prosperity without which mere political changes are of little use. Let politicians of every shade of opinion get their just meed of praise for whatever honest endeavour they have shown, but even if they are forgotten, one and all, let the name of Mrs. Ernest Hart be written in gold when the book of the making of Ireland shall come to be compiled.

**Molokai in Calcutta.**

A few days ago the *Daily News* contained an account of a scene in the streets of Calcutta. Miserable lepers, seated in a light cart, were driven through the streets, drawn by a relative or a starveling pony. They sought alms, but the manner in which they drew their ragged clothing over their sores indicated a fear lest the compassion they sought to excite might change into disgust. Those miserable creatures do not frequent the best parts of the town, and many of the European inhabitants are in total ignorance of the number of lepers in their midst. That means that the dominant class do not, as a rule, know how many sufferers from a contagious disease dwell within a mile of them with no precautions against their infecting others. This ignorance can hardly be regarded as other than reprehensible. It is contrary to all human feeling to let incurable sufferers be totally dependent on a precarious charity, or even to have their misfortunes farmed as a revenue by unscrupulous relatives. It is equally contrary to the principles of wise administration to let a source of contagious disease of any kind run loose among the populace. There may be many things in the way of drugs and treatment that may alleviate the condition of the individual leper; but as a national disease there is only one cure—isolation. In Norway, isolation has been recommended for many years, and has been insisted on only since 1885. The result is that leprosy is steadily decreasing. The number of lepers in Norway is about 1,000, less than half what they were when restrictive methods were introduced thirty-three years ago. The isolation law is not by any means a cruel one according to the statement of Dr. Robson Roose, who says: "A leper, not an inmate of a hospital, must confine himself to one room in the house which he or his friends occupy. He may take exercise in the open air, and be accompanied by his friends or other persons; but he must not enter any other dwelling-house, or any room in his own house, save the one apportioned to him. Compulsory removal to a special hospital and detention therein are the penalties attached to any infringement of these regulations." Residence in a special hospital is indeed always to be preferred for the patient's sake, since there the alleviations possible to medical science are at hand; but this isolation, which does not wholly destroy the bond of family life, is all that is insisted on. That a wife should be permitted to share her husband's solitude if she will, is a point which all Lord Tennyson's genius will hardly make us concede. But whatever modifications humanity and expediency may suggest, it is surely time that the Government of India laid down as a first principle of sanitation the compulsory isolation of lepers.

**A St. Thomas' Dresser.**

Many readers will already have seen the account of the death of Mrs. Elizabeth Reynard, on whose body an inquest was held by Mr. G. P. Wyatt last week. Mrs. Reynard was a dresser at the Vaudeville Theatre. She had had an attack of the prevalent influenza, followed by bronchitis, and during her illness had fainted more than once. Having returned to her duties at the theatre before her strength was restored, Mrs. Reynard on the very first evening had several times complained to a friend of feeling unwell. She left the theatre a little before midnight, and probably still feeling weak and ill, had purchased and taken two pennyworth of gin. Before one o'clock she was found on the centre landing of the steps of Waterloo Bridge, bleeding and partly unconscious. She was then conveyed to St. Thomas' Hospital, where she was seen and attended to by Mr. William Thurman, one of the dressers on duty at the time. The dresser considered that Mrs. Reynard was partly intoxicated, and in spite of a wound in the head, which afterwards proved to be connected with a fracture at the base of the skull, ordered her to be taken away. She was taken away, and treated by the police throughout the night as a drunken woman, although it was proved in evidence that she was very abstemious. Needless to say, Mrs. Reynard shortly died, and that without having recovered consciousness. It was the dresser's duty, according to the rules of the hospital, to have called the house surgeon; but he, a young, most probably unqualified, and apparently quite inexperienced man, took upon himself to disregard this rule, with consequences such as have been described. The jury returned a verdict, with a rider, in which they very mildly censured Mr. Thurman. It is to be hoped, however, that he himself takes a much more just view of the circumstances. Young doctors and medical students often seem to fail entirely to understand the deep and serious responsibility of the duties committed to their charge. In this poor woman's case character and life were both at the dresser's mercy, and how terribly he failed to appreciate the gravity of the case is all too plainly evident. If his conscience does not convince him of the unworthy nature of his conduct, nothing that we can say will have that effect. It may be hoped, however, that other dressers and house surgeons will take this unhappy story to heart. The hospital authorities, so far as we can see, are entirely without blame in the matter.

